# Medium‐term outcomes of hybrid total hip arthroplasty in cats: Cemented femoral stem and cementless acetabular cup in 17 hips (2020–2023)

**DOI:** 10.1111/vsu.14274

**Published:** 2025-06-05

**Authors:** Daniel Lomas, Sorrel Langley‐Hobbs, Kevin Parsons, Nicolas Barthelemy

**Affiliations:** ^1^ Langford Veterinary Services Bristol UK; ^2^ Faculty of Health Sciences, Bristol Veterinary School University of Bristol Bristol UK

## Abstract

**Objective:**

To describe the surgical technique, complications and outcome of hybrid total hip replacement (THR) in cats using a BioMedtrix cemented femoral stem and cementless acetabular cup.

**Study design:**

A retrospective case series.

**Animals:**

Cats undergoing hybrid THR between 2020 and 2023.

**Methods:**

The present study was a retrospective review of medical records. Postoperative radiographs were reviewed and implant positioning measured. Owner‐reported follow‐up was obtained via questionnaire utilizing the short‐form feline musculoskeletal pain index (sf‐FMPI).

**Results:**

A total of 17 hybrid THRs were performed with two cats undergoing bilateral THR. A 12 mm BioMedtrix micro BFX acetabular cup was placed in all cats in combination with a CFX femoral stem. Follow up imaging demonstrated no change in implant position. No intra‐ or postoperative complications were reported. Hybrid THR was successfully performed in one case as a revision strategy for recurrent hip luxation following CFX THR. Owner‐reported follow‐up was obtained for 10 out of 15 cats (mean 438 days postoperatively; range, 185–1084 days). Overall satisfaction was excellent and the mean sf‐FMPI score was 2/36.

**Conclusion:**

This study demonstrates that hybrid THR using a micro BFX acetabular cup is feasible in cats with a good medium to long‐term outcome. These results compare favorably to previous reports of feline THR.

**Clinical significance:**

Hybrid THR in cats has a low complication rate and high owner satisfaction. This technique should be considered for the management of coxofemoral disease in cats and can be considered for CFX cup revision.

## 1 INTRODUCTION

Total hip replacement (THR) is indicated as a salvage procedure for numerous conditions affecting the feline hip joint including hip dysplasia, osteoarthritis, slipped capital femoral epiphysis (SCFE), articular fractures of the femoral epiphysis, recurrent luxation and revision of unsuccessful femoral head and neck excision (FHNE).^1^, ^2^, ^3^, ^4^ A recent large cohort study of cats undergoing THR demonstrated an overall complication rate of 19.6% (11/56) with 90.9% of owners considering the outcome “very good”.^4^ In addition, a short case series comparing the outcomes of three feline THRs to femoral head and neck excision demonstrated improved thigh girth, hip range of movement, and functional outcomes in the cats who underwent THR.^5^ This was further corroborated by Witte and colleagues who reported on five feline THR including one case which underwent unilateral THR and contralateral FHNE.^6^ This case was demonstrated to have a reduced thigh circumference on the FHNE side suggesting chronic offloading of this limb.

Hybrid THR, which combines cemented and cementless systems, has been reported to have lower long‐term complication rates in medium and large‐breed dogs compared to cemented and cementless systems.^7^, ^8^ Most commonly a cemented femoral stem is combined with a cementless acetabular cup. Micro BFX acetabular components have recently become available on the market allowing the option of hybrid THR in patients under 12 kg which would have previously been limited to cemented THR. This may provide a significant advantage in feline THR where achieving an adequate cement mantle around the acetabular component can be particularly challenging.^2^ Although the majority of reported feline THRs are using cemented systems, the Zurich mini cementless total hip arthroplasty has recently been described in eight cats.^9^

The objective of this study was to describe the medium‐term outcomes of hybrid THR in cats, using a CFX BioMedtrix femoral stem in combination with a BioMedtrix micro BFX acetabular cup. The study examines complication rates and owner‐reported outcomes and represents the first description of hybrid THR and the use of the BioMedtrix BFX acetabular cups in cats.

## 2 MATERIALS AND METHODS

Medical records between 2020 and 2023 from a single referral institute were retrospectively reviewed for cats undergoing total hip replacement (THR). The primary inclusion criteria were cats presenting with coxofemoral disease who subsequently underwent THR using a hybrid system. Complete clinical records were required and short‐term radiographic follow‐up at 4–6 weeks was preferable. The local research ethics review committee granted ethical approval for the study (VIN‐23‐023).

Recorded information included signalment (age, breed, sex, neuter status), bodyweight and history of prior surgery. Mode of preoperative imaging and radiographic diagnosis was recorded. Contemporaneous surgical data included implant sizing, additional procedures, intraoperative complications and mode of immediate postoperative imaging. Follow‐up imaging at 4–6 weeks was reviewed. Clinical records from the revisit appointment and primary care veterinarian were reviewed for any additional complications.

Preoperative radiographs or computed tomography (CT) were obtained under sedation for the purpose of preoperative planning. For patients undergoing radiography, preoperative projections included a ventrodorsal and mediolateral view of the pelvis along with a mediolateral and craniocaudal view of the femur on the affected side. Horizontal beam positioning was utilized for craniocaudal projections of the femur with care taken to ensure the femur was parallel to the X‐ray plate and that the fabellae bisected the femoral cortices to ensure a true craniocaudal projection. Superimposition of the femoral condyles was required for the lateral view of the femur. Symmetry of the iliac wing and obturator foramen on the ventrodorsal view of the pelvis and superimposition of the iliac wing and ischial tuberosity for the lateral view of the pelvis. For patients which underwent CT assessment preoperatively, multiplanar reconstruction (Horos, version 3.3.6.; Horos Project, Geneva, Switzerland) was utilized to create orthogonal views of the pelvis and femur recreating the radiographic projections. The mean function was used and slice thickness increased until the appearance of the radiograph was achieved, and a reference line added for calibration of the image in the templating software. Radiographs and CT images were imported into templating software (VPOPpro Column House, Shrewsbury, UK) for surgical planning.

Cats were anesthetized using an appropriate protocol as determined by a board‐certified anesthetist. All cats received perioperative analgesia including opioids at the time of induction and repeated intraoperatively as required. All cats received a lumbosacral epidural with morphine sulfate (0.1 mg/kg, Hameln Pharma Ltd., Gloucester, UK) and bupivacaine hydrochloride (1 mg/kg, Aspen, Dublin, Ireland). Perioperative antibiotics were administered 30–60 min prior to first incision and repeated every 90 min intraoperatively (cefuroxime 20 mg/kg IV, Sandoz AG).

Cats were positioned in lateral recumbency using a Vac‐Pac positioning aid (Olympus Vac‐Pac Positioning Aid, Freelance Surgical, UK) and secured with tape. Preoperative fluoroscopy was used to ensure correct patient positioning, and that the patient was in straight lateral recumbency with superimposition of the lumbar transverse processes, ilial wings and ischiatic tuberosities. The limb was draped with adhesive four‐quarter draping followed by an impervious U‐drape. An adhesive incision drape was then used (Ioban, 3M UK PLC). After skin incision an impervious drape was stapled to the skin edges to ensure the skin edges were everted and covered for the entire procedure. A routine craniolateral approach to the hip joint was performed including a partial tenotomy of the deep gluteal tendon. The hip was luxated and a femoral head and neck ostectomy performed at the appropriate level identified on preoperative surgical planning and by use of the resection guide. A Steinman pin was manually positioned by an assistant at the level of the dorsal iliac wing cranially and ischiatic tuberosity caudally to obtain the alignment of the pelvis. This allowed the primary surgeon to assess the inclination of the acetabulum while using the small‐breed reamer alignment guide.

The acetabulum was initially reamed using a 10 mm reamer and then followed by the 12 mm reamer to allow placement of a 12 mm acetabular component. Once available 9 mm and 11 mm reamers were used in addition to the 10 mm and 12 mm. A finishing reamer is not available for the micro BFX acetabular cup. A 12 mm trial acetabular cup was used to evaluate cup depth and positioning. A 12 mm, internal diameter (ID) 8 mm, BioMedtrix micro BFX acetabular cup (BioMedtrix, Whippany, New Jersey) consisting of a Poly‐Xve liner (highly crosslinked and vitamin E stabilized ultra‐high molecular weight polyethylene) and electron beam melting titanium cup with a porous outer surface was inserted into the reamed acetabular bed and the positioning adjusted. Care was taken to ensure no soft tissue was trapped between the implant and the acetabulum through the use of Hohman retractors, Gelpis and a Freer periosteal elevator on the ventral acetabular border. The cup was impacted using the 12 mm concentric impactor with the aim of matching the native acetabular anatomy. When the native anatomy was significantly modified, the aim was to place the cup at an angle of 20° of inclination and 10° of retroversion and with an angle of lateral opening of 45°–55°. Inclination was assessed by using a Steinmann pin as described for assessing alignment of the reamer and by palpating the lumbar spine with the aim being that the points of the truncated surface were roughly parallel to the lumbar spine and 20° to the pin. Cup retroversion was assessed by comparing the position of the cup in relation to the cranial and caudal borders of the acetabulum. To achieve the desired angle of lateral opening the truncated surface of the cup was positioned parallel to, or with a slightly ventrally directed slope, compared to the surgical table. Positioning was adjusted using a combination of the eccentric impactor and the version guide rod.

The femoral medullary canal was accessed using either Steinmann pins of increasing size or a hand awl at the craniolateral intertrochanteric fossa, depending on surgeon preference. The femoral canal was then prepared using a combination of power reaming, filing, and broaching until the femoral stem could be placed with allowance for an adequate cement mantle. This was assessed using a trial stem. In several cases the hip was reduced with the trial implant and tension assessed, the main aim being to confirm that the hip could be reduced, as if this was not possible the osteotomy of the femoral neck could be repeated to distalize the stem. Low viscosity polymethylmethacrylate bone cement with gentamicin (Eurofix veterinary bone cement with gentamicin: low viscosity, Securos UK) was prepared in a mixing bowl. Pulsatile lavage and suction were used to clear fat and blood from the femoral medullary canal and dry the bone surface. Once the desired consistency was achieved, bone cement was injected into the cavity using a 10 mL syringe and a 10 fr rigid urinary catheter trimmed to length with a steeply angled cut. The stem was inserted and held in the desired neutral or mildly anteverted position until complete setting of the bone cement.

A trial head was placed (8 mm +0) and the hip reduced. The arthroplasty was examined throughout a full range of movement and assessed for complementary implant positioning and implant impingement. At this stage, should the implant positioning be unsatisfactory, the cup position could be adjusted. The fully seated cup was removed by applying controlled impacts with a mallet, using either the eccentric impactor or the version guide rod positioned at the dorsal (12 o'clock) aspect of the cup. Alternatively, a thick, blunt osteotome was used to strike the porous dorsal surface of the cup at approximately the 12 o'clock position, directing the force from dorsal to ventral to dislodge the implant. The cup was repositioned either by complete removal and replacement or by adjusting the position without removal. The same implant was used in all cases and the impaction process repeated. The same implant was used in all cases and the impaction process repeated. Soft tissue tension was assessed using the claw portion of a Senn retractor on the greater trochanter. With the hip in neutral position, gentle laterally directed tension was placed and displacement of the femoral head assessed. Tension was deemed appropriate if the cat's pelvis could be slightly lifted without hip luxation. If excessive soft tissue laxity was identified the head offset was increased as required by placement of a + 2 trial head. The procedure was repeated with consideration given to the use of a + 5 femoral head if required. The trial head was then replaced with a cobalt chrome femoral head of the corresponding neck length.

The surgical site was lavaged with sterile saline prior to routine closure in layers. The joint capsule was closed with 2–0 USP polydioxanone (Ethicon, Johnson and Johnson, Capitol Park, Leeds, UK) in an interrupted cruciate pattern. Closure of the deep gluteal tenotomy was performed using 2–0 USP polydioxanone in a modified continuous cruciate pattern similar to THR in dogs. The remainder of the incision was closed in layers.

Cats were hospitalized for 1–2 days post‐surgery and received both opioid analgesia and non‐steroidal anti‐inflammatories. Cats were discharged with a further 7–14 days of meloxicam (0.05 mg/kg orally every 24 h) +/− gabapentin (10 mg/kg orally every 8 h). Postoperative antibiotics (cephalexin 20 mg/kg orally every 12 h) were dispensed for 5–7 days. Postoperative care instructions recommended strict crate confinement for 6 weeks. For unilateral THR cases follow‐up imaging was performed at approximately 6 weeks postoperatively with either radiographs or CT depending on surgeon preference. For planned bilateral THR follow‐up imaging was performed at 4 weeks prior to the contralateral THR and then again at 6 weeks post the second side. Images were assessed for complications such as bony changes, implant loosening, migration or dislocation and presence of radiolucency at the bone‐cement or bone‐implant interface. In the absence of complications, a gradual increase in exercise was recommended which included 2 weeks of room confinement without furniture, 2 weeks of confinement to a room with low furniture, 2 weeks of free roam of the house when supervised then a full return to freedom including outdoor access.

Immediate postoperative imaging was assessed for implant positioning. Angle of lateral opening (ALO) was estimated by assessing the truncated surface of the acetabular cup on the ventrodorsal view and recorded in 10° increments.^10^ When the truncated surface appeared as a straight line then the ALO was recorded as 40°. A mildly convex surface was recorded as an ALO of 30° and 20° if markedly convex. Conversely, mildly and markedly concave was recorded as 50° and 60°, respectively. Inclination was measured on the lateral view by drawing a line bisecting the most cranial and caudal points of the truncated portion of the cup and a line marking the ilial‐ischial axis.^11^ The angle between the two lines represented the angle of inclination. Version was measured on the ventrodorsal view using a line parallel to the transected portion of the cup and a line parallel to the pubic symphysis with the angle between the two recorded.^12^ Additionally, on the ventrodorsal view, the magnitude of any polar gap between the medial aspect of the cup and the bone was measured at its widest point.

Postoperative complications were recorded as catastrophic (permanent unacceptable function requiring implant removal, death or euthanasia), major (required surgical intervention to resolve), and minor (did not require surgical treatment).^13^ If >6 months had elapsed since the initial surgery the cats' primary care veterinarian was contacted, and any complications not already recorded in the hospital records were noted. Owners were contacted and asked to complete the feline musculoskeletal pain index (short version) (PDF in supplementary information) with a minimum of 3 months post‐surgery which included all cats. This time frame was chosen as based on the exercise plan this would represent a full return to freedom. Frequencies for nominal data are described. Variables are reported as mean and range.

## 3 RESULTS

A total of 17 hybrid THRs were performed in 15 cats during the study period, with two cats undergoing staged bilateral THR. The mean age at surgery was 24 months (range, 14–128 months). The mean weight was 5.2 kg (range, 7.6–3.5 kg). There were 13 male neutered cats and two female neutered cats. Breeds were British Shorthair (*n* = 5), Domestic Longhair (*n* = 3), Domestic Shorthair (*n* = 3), Bengal (*n* = 2) and Maine Coon (*n* = 2). The indication for THR in 13/17 cases was SCFE. Both cats who underwent bilateral THR had bilateral SCFE. For the remaining four cases THR was performed due to hip dysplasia (*n* = 1), traumatic luxation and subsequent failure of hip toggle (*n* = 1) and a traumatic comminuted femoral neck and avulsion of the greater trochanter (*n* = 1). The remaining cat underwent hybrid THR to revise a CFX THR. The initial surgery had been performed due to chronic lameness post‐FHNE. Revision of the CFX acetabular cup to a BFX was performed due to recurrent luxation. Preoperative imaging was performed in all cats with six cats undergoing preoperative CT and radiographs in the remaining nine cats.

A size 2 CFX femoral stem was placed in one case. For the remaining 16 cases, a size 3 CFX femoral stem was placed. No intraoperative complications occurred during stem placement. A 12 mm BFX acetabular cup was placed in all cases. Satisfactory press fit was achieved in all cases. A size 8 mm +0 femoral head was placed in 13/ 17 cases. A size 8 mm +2 femoral head was placed in 4/17 cases. Head offset was based on intraoperative assessment of soft tissue tension. After reduction of the prosthesis, a tendency for medial patella luxation was identified in three cats. Stifle flexion was also reduced in these cases. This was deemed secondary to soft tissue tension in the quadriceps. A partial tenotomy of the origin of the rectus femoris was performed in these three cases resulting in a resolution of medial patella luxation and an improvement in the stifle range of flexion. Two of these cases had a + 0 mm femoral head placed and one had a + 2 mm femoral head. A pin and tension band were placed to stabilize a concurrent avulsion of the greater trochanter in the cat with the preoperative comminuted femoral neck fracture. In the latter cat preparation of the femur was performed routinely. Once the stem had been placed and the hip reduced the greater trochanter was reduced and stabilized with a pin and tension band as would be performed when repairing this injury in isolation. The Kirshner wires were placed from proximolateral to distomedial with an attempt to avoid the stem. One case underwent revision of a previous CFX THR due to recurrent luxation. In this case a standard surgical approach was made and the femur retracted caudally to expose the acetabulum. The previously placed cup and bone cement were removed using a combination of rongeurs, osteotome/mallet and high speed burr. In addition, the SOP plate which had been placed to augment the dorsal acetabular rim was removed. Once all of the cement had been removed reaming was performed using the standard reamers described above. Reaming was directed slightly dorsally and continued to the medial acetabular wall to ensure exposure of cancellous bone and achieve sufficient dorsal coverage. Two cats underwent repositioning of the acetabular cup after stem placement due to malalignment of the prostheses.

All cats were discharged within 48 h postoperatively and deemed to have satisfactory use of the operated limb by the attending clinician. Re‐examination and radiographic follow‐up were performed at a mean of 41 days postoperatively (range, 24–55). Limb use was assessed and no lameness was reported in 11/15 cats. Four cats were reported to have a mild lameness in the operated limb. Follow‐up imaging was performed in 14 out of 15 cats at the initial recheck, confirming that implant position was unchanged, and no additional complications were identified. CT was performed as the sole follow‐up imaging in three cats with radiographs performed in the remainder. The cat which did not undergo 6‐week radiographic follow‐up re‐presented 12 months later with a contralateral SCFE. Radiographs performed at this stage revealed implants unchanged in position due to successful osteointegration.

Primary care veterinarians were contacted for follow‐up at the time of preparing the patient manuscript. No additional complications were reported with long‐term follow‐up (>365 days) available for eight out of 15 cats (mean 474 days postoperatively, range, 168–1174). Owner reported follow‐up was obtained for 10 out of 15 cats at a mean of 438 days postoperatively (range, 185–1084 days). The mean FMPI‐sf score was 2/36. A score of 0/36 was obtained for four cats. Two cats scored 1/36. The remaining four cats scored 3, 5, 7 and 9. The cats with a score of 5 or greater all scored 0 in all questions except for the two questions related to jumping up to a height. One of these cats presented 12 months after the original THR and 5 months post completing the questionnaire with a contralateral SCFE.

### 3.1 Radiographic measurements

Evaluation of the immediate postoperative radiographs or CT revealed satisfactory press fit with a 1 mm gap identified in one case, 0.5 mm in 5/17 hips and no gap in the remaining 11 hips. The mean angle of lateral opening was 38.2° (range, 30°–50°). The mean cup inclination was 25.8° (range, 15.1°–36.9°). The mean cup version was 7.7° (range, 0.5°–18.4°) (Table 1).

**TABLE 1:** Patient data including age, breed, sex, neutering status, implants used and acetabular cup position.

Patient	Side	Age (months)	Breed	Sex	Weight (kg)	CFX stem	Head diameter (mm)	Head offset (mm)	BFX cup size (mm)	Cup ALO (°)	Cup inclination (°)	Cup version (°)
1	Left	17	DMH	FN	4.3	2	8	0	12	30	32	9.4
2	Left	30	DMH	MN	6.2	3	8	0	12	40	24	10.1
3	Right	17	BSH	MN	5.5	3	8	0	12	30	22	8.1
3	Left	18	BSH	MN	5.5	3	8	0	12	50	25	16.4
4	Left	24	BSH	MN	6.7	3	8	0	12	30	23	0.5
5	Right	75	DSH	MN	4.2	3	8	2	12	30	20	5.8
6	Left	15	Bengal	MN	4.9	3	8	0	12	50	26	14
7	Right	29	DSH	MN	4.8	3	8	0	12	30	36.9	1.3
8	Left	20	Maine Coon	MN	7.6	3	8	0	12	30	25	5.6
9	Right	84	Maine Coon	MN	4.1	3	8	0	12	30	17	^a^
10	Left	127	BSH	FN	4.4	3	8	2	12	50	34	1
10	Right	128	BSH	FN	3.9	3	8	0	12	30	30	8.9
11	Right	24	BSH	MN	5.6	3	8	0	12	40	15.1	3.9
12	Left	14	DLH	MN	3.5	3	8	2	12	50	27.5	18.4
13	Right	18	DLH	MN	4.7	3	8	0	12	40	27.9	8.3
14	Left	39	BSH	MN	6.6	3	8	0	12	50	28.6	10.9
15	Right	19	Bengal	MN	5.2	3	8	2	12	50	25	0.7

*Note*: Grant: Project no AOVETS‐24‐03 L was supported by AO Foundation, AO VET. AO VET is a clinical division of the AO Foundation – an independent medically‐guided not‐for‐profit organization, Switzerland.

Abbreviations: BSH, British Shorthair; DLH, Domestic Long hair; DMH, Domestic medium hair; DSH, Domestic Short hair; FN, female neutered; MN, male neutered.

^a^ Unable to measure version on ventrodorsal view due to distortion of normal anatomy due to previous pelvic fractures.

## 4 DISCUSSION

The present study demonstrates that hybrid THR using a micro BFX acetabular cup (12 mm ID8 mm) is feasible in cats with a good medium to long‐term outcome. A recent large cohort study of cats undergoing THR with cemented implants reported an overall complication rate of 19.6%.^4^, ^7^ There were no major postoperative complications noted in our study. A previous study in dogs has also reported a lower complication rate associated with hybrid THR when compared to CFX and BFX THR.^8^

No intraoperative, immediate postoperative or medium‐term complications were reported in our small cohort of cats undergoing hybrid THR. In the largest report of feline THR, luxation was the most frequent major complication, occurring in 10.7% of cats at a median of 26.5 days postoperatively.^4^ A lower rate of luxation was observed in hybrid THR in dogs when compared to CFX and BFX THR.^7^ Complementary position of THR implants is desirable to avoid implant impingement and subsequent luxation. One main intraoperative advantage of the hybrid THR is the ability to reposition the acetabular cup both pre‐ and post‐stem implantation. Due to the retrospective nature of this study, details regarding cup repositioning were not recorded; however, discussion between the surgeons involved in this cohort revealed that in two cases the cup was repositioned due to impingement noted after hip reduction.

Another reason for the absence of postoperative luxation in this cohort is that the global offset of the hip may have been maintained or even increased. Global offset is defined as the combined acetabular and femoral offset.^14^ Acetabular offset is influenced primarily by cup size and positioning and refers to offset of the center of rotation of the acetabulum from the medial wall of the acetabulum. Femoral offset refers to the distance of the femoral head from the anatomical axis of the femur and is influenced by the head offset options available (+0, +2, and + 5 mm). Anecdotally, the 12 mm BFX acetabular cup in the cat appears to sit more prominently relative to the acetabulum compared to a CFX acetabulum. In addition, our early cases had cups implanted in a more lateralized position, with some cases utilizing a cup that may have been considered oversized (12 mm). This may have contributed to an increase in acetabular offset.

This speculated increase in acetabular offset seems supported by our data showing that the +0 head offset on the 8 mm head was the most frequently used (13 out of 17 cases). This contrasts with previous findings, where the +2 mm head offset was more commonly selected.^4^ Rodiño Tilve et al. suggested that a longer neck (+2 on an 8 mm head), which would increase femoral offset, could help reduce the risk of luxation.^4^ Our findings do not support this, but if we consider global offset rather than acetabular or femoral offset alone, it is possible we achieved a similar effect by increasing the acetabular offset rather than the femoral offset.

In this cohort, three cats underwent a partial tenotomy of the tendon of origin of the rectus femoris to address medial patella luxation. Medial patella luxation (MPL) has previously been reported as a complication of THR in cats.^4^ Failure to correct this may require corrective surgery at a later date and has been hypothesized to be linked to femoral offset resulting in lateralization of the femur and a resulting increase in tension in the rectus femoris muscle.^15^ In this cohort, a visible reduction in soft tissue tension and a resolution of medial patella luxation were found after a partial tenotomy of the rectus femoris tendon of origin. No cases of postoperative patella luxation were seen and no cats required more conventional corrective procedures such as block sulcoplasty and tibial tuberosity transposition. This technique has not been described before and may offer an option for the management of MPL associated with feline THR; however, this must be interpreted with caution due to the low numbers. No increase in lameness was reported in the cases that underwent this procedure and two of the three had low sf‐FMPI scores. The one cat who scored higher had undergone bilateral THR.

Implant positioning of feline THR has been based on that for canine THR. The recommended angle of lateral opening (ALO) for canine THR is 35°–45° and version of 15°–25°.^11^ These angles have been extrapolated to cats. The mean ALO in this study was 38.2°; however, several acetabular cups were positioned outside the recommended range of 35°–45°. Despite this, no cases of luxation occurred within this limited cohort. The reported cup orientation angles are provided primarily as reference values, and any interpretations should be approached with caution. Further studies involving a larger sample size are warranted to better establish a range of cup orientation angles that may reduce or increase the risk of complications in cats. In this cohort, complimentary positioning of the acetabular and femoral component was prioritized. Ensuring a lack of impingement throughout a full range of movement and satisfactory soft tissue tension may have contributed to the lack of luxation despite the relatively large range of implant positions. In addition, there is no standardized technique for measuring the positioning of the 12 mm BFX cup. The technique used was a modification of a previously published reference table.^10^ Due to the relatively small proportion of the truncated surface visible with a 12 mm cup it is possible that this technique is less reliable when applied to this size of implant. In addition, several publications have examined the limitations of assessing ALO on the ventrodorsal view and through use of the truncated.^16^, ^17^

Subjectively, feline acetabular anatomy provides more limited bone stock for cup implantation compared to canine anatomy, making it challenging to achieve an adequate cement mantle for CFX implants. This challenge is even greater in cases where part of the dorsal rim has been lost such as cases where THR is performed due to a poor outcome post FHNE.^2^ Despite this reduced bone stock in cats, no difficulties were encountered during BFX cup implantation, and no cases of early implant loosening were observed. Although the 12 mm acetabular cup allows the use of a modular femoral stem and head, it appears subjectively large for some of the cats. Despite this, no cases of cup dislodgment were noted in this cohort. Stability of BFX acetabular components has been demonstrated with 50% loss of the dorsal acetabular rim.^18^ Our results suggest that a similar level of stability is seen in micro BFX cups when used in cats with reduced acetabular bone stock.

**FIGURE 1:**
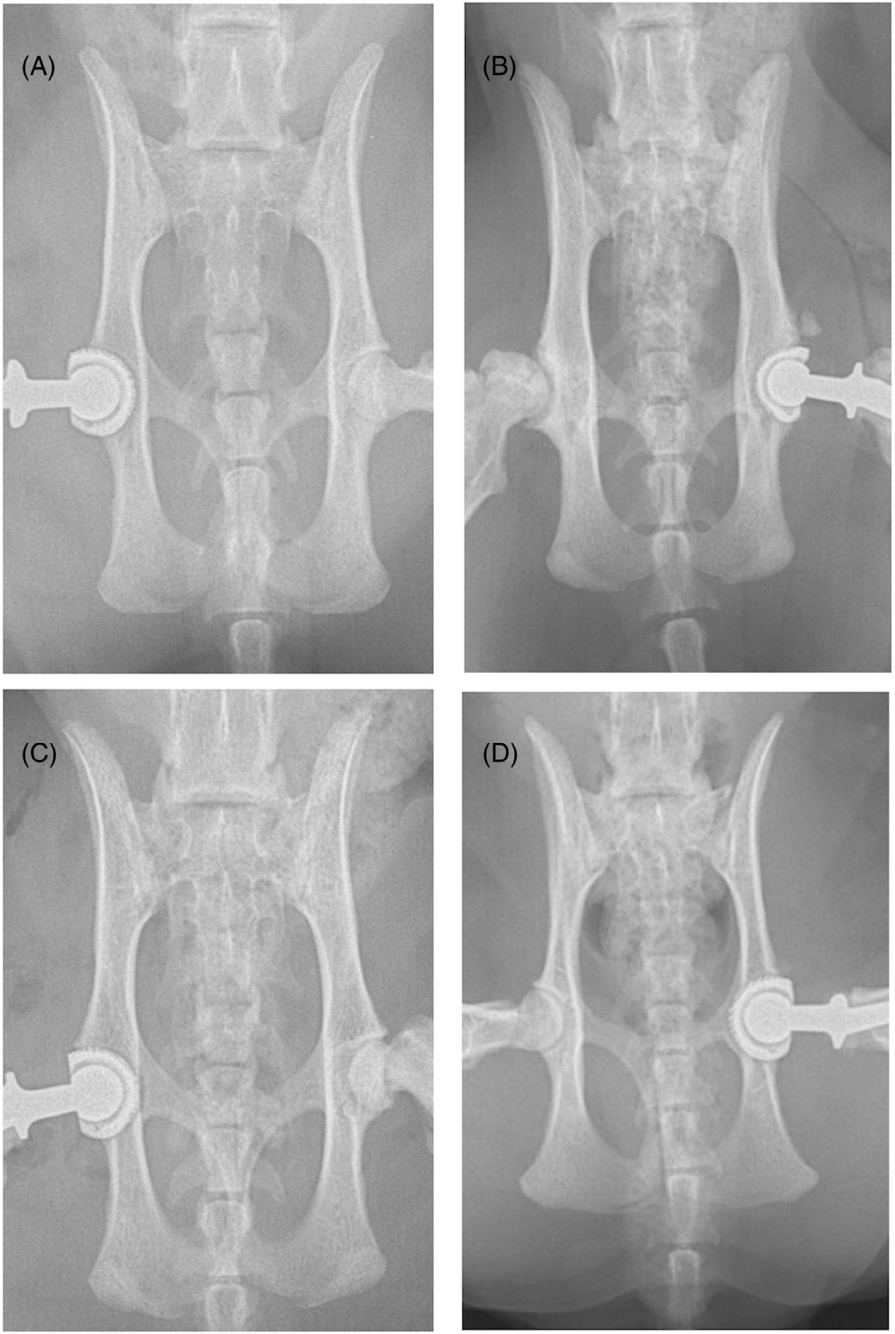
Postoperative ventrodorsal radiographs demonstrating cup position. (A and B) A 12 mm BFX acetabular cup placed in a lateralized position. (C and D) The acetabular cup placed more medially with involvement of the medial acetabular wall.

In the initial cases receiving hybrid THR, the surgeons lacked experience with the stability of the micro BFX cup, and the necessary ancillary equipment was only available on a loan basis, limiting options for revision with another BFX cup. As a result, acetabular preparation aimed to avoid excessive medialization, so a CFX cup could still be placed if revision surgery was required. This approach led to generally shallower seating of the cup within the acetabulum, with reduced dorsal coverage (Figure 1A,B). Despite this, no cases of early implant loosening were encountered. As experience with the technique increased and the micro BFX kit became permanently available, the approach evolved to prioritize optimal dorsal coverage, including, in certain cases, partial thickness or complete breaching of the medial wall to enhance cup stability (Figure 1C,D). Use of the 12 mm cup combined with an 8 mm head was preferred as it provided the opportunity to adjust soft tissue tension by use of a + 2 mm or + 5 mm head and the desired neck offset compared to a Monoblock stem (6 mm head). This resulted in several cups which could be considered slightly oversized; however, this did not appear to affect their stability.

**FIGURE 2:**
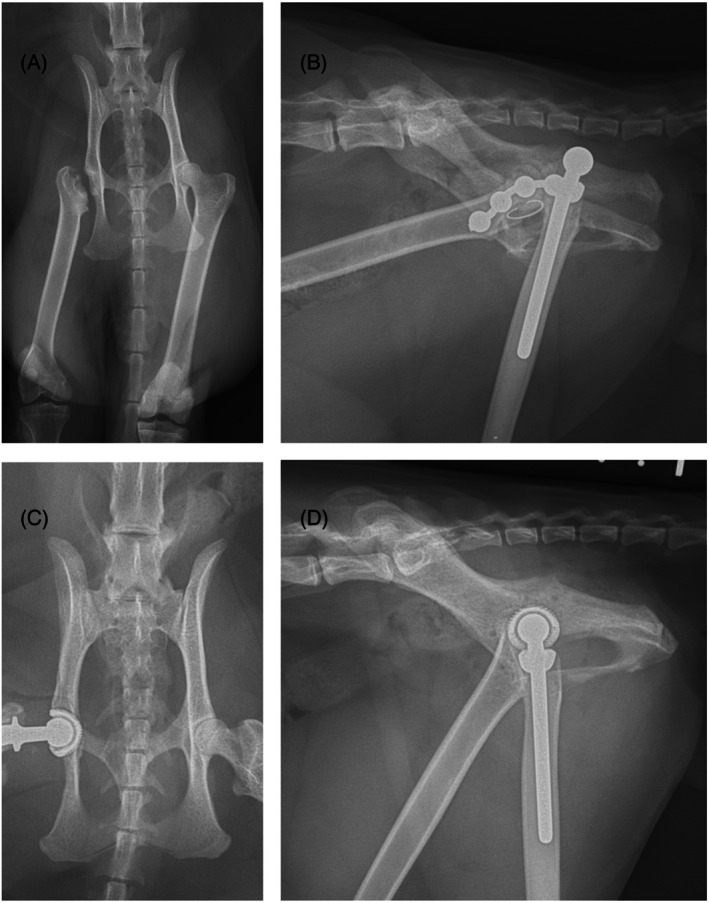
(A) 1‐year‐5‐month‐old male neutered Bengal presented due to lameness post femoral head and neck excision. (B) Caudo‐dorsal luxation of CFX total hip replacement (THR) with augmentation of the dorsal acetabular rim with a string of pearls plate. (C and D) Revision of the CFX THR with a BFX acetabular cup.

One case underwent revision surgery using a micro BFX cup for recurrent luxation of a previously placed CFX cup. This cat initially had limited acetabular bone stock due to a prior FHNE, and at the time of the initial surgery, the BFX cup was unavailable (Figure 2A). Given the absence of the dorsal rim and the reported risk of aseptic loosening the initial surgery used a 12 mm CFX cup with dorsal rim augmentation using a string of pearls (SOP) plate (Orthomed, Huddersfield, UK).^2^, ^19^ Despite this, the cat experienced two luxation recurrences (Figure 2B). During the final revision, both bone cement and the dorsal plate were removed, and the BFX cup was successfully implanted despite the limited remaining bone stock (Figure 2C,D). Radiographic follow‐up at 6 weeks and mid‐term owner feedback were positive, with no further luxation and stable implant fixation. Although this is a single case, it suggests that hybrid THR may be a viable revision option for failed CFX THR in cats.

**FIGURE 3:**
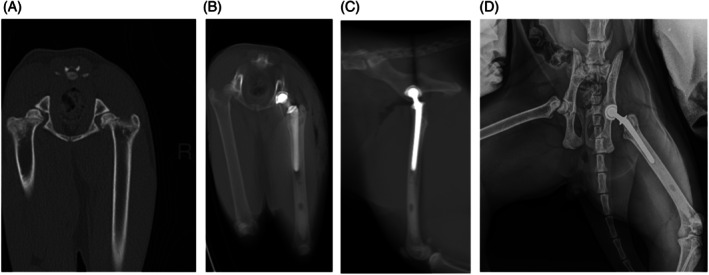
(A) 2‐year‐old male neutered British Shorthair presented with a right slipped capital femoral epiphysis (SCFE). (B and C) Right hybrid total hip replacement using a 12 mm micro BFX acetabular cup and a size 3 CFX femoral stem with an 8 mm +0 head. (D) Twelve months later, the cat presented with left hindlimb lameness, and radiographs demonstrated a left SCFE and stable implants on the right, indicating osseointegration.

Overall, the clinical outcome for this technique was considered very good. All cats were consistently weightbearing at 24 h postoperatively. In the author's experience, similarly to small and miniature dog breeds, cats are more mobile than large dogs at this stage postoperatively. This can make enforcing postoperative confinement more challenging. At approximately 6 weeks postoperatively the majority of cats were assessed as having no lameness, and four cats were found to have a mild lameness. Owner reported follow up at a mean of 403 days (range, 185–1084) postoperatively demonstrated most cats had returned to what their owners considered a normal level of activity. The three cats with a score >5/36 were reported to have difficulty jumping onto high surfaces. Jumping is likely to require significant force and full flexion followed by rapid extension of the hip joint. Of these cats, one represented 6 months after the owner completed the questionnaire with a contralateral SCFE, questioning whether this could have contributed to the higher score, and one underwent bilateral THR (Figure 3). It is, however, possible that the biomechanics of feline THR could impact the ability to jump. Due to the low case numbers further investigation of the impact of implant positioning on postoperative outcome was not possible.

Slipped capital femoral epiphysis was the most common indication for THR in this study. Consistent with the typical signalment of this condition, most cats in this cohort were juvenile (median age 24 months), neutered male cats. The young age of the cats in this study and their expected life expectancy means that the implants are anticipated to have a long working life. The maximum radiographic follow‐up in this study was 12 months (one cat) and therefore long‐term stability of BFX acetabular components in cats is currently unknown. Long‐term implant stability has been demonstrated in hybrid THR in dogs.^10^ Further investigation in cats should include lifelong radiographic follow up and postmortem biomechanical testing to determine if these implants withstand the rigors of a cat's activity.

There were several limitations to this study. The low case numbers increase the risk of drawing incorrect conclusions. Radiographic follow‐up was performed at a mean of 41 days postoperatively (range, 24–55) and therefore long‐term stability of the implants or associated remodeling could not be fully assessed other than in one cat which re‐presented 12 months after the initial surgery with a contralateral SCFE. Long term follow‐up was done via an owner questionnaire. There are several limitations to owner‐assessed outcomes in cats. Witte and colleagues^6^ reported one case where, at 15 weeks post THR, the owners reported good outcomes; however, radiographs revealed a craniodorsal luxation of the prosthesis.^6^ Owner and vet assessment has been demonstrated to be poor in detecting small changes in ground reaction force in cats after femoral head and neck excision.^20^ Objective gait analysis would have allowed improved outcome assessment in this cohort of cats. The technique utilized for measuring ALO in this study is not validated. The ellipse template estimates ALO using trigonometry but due to the truncated surface of the BFX acetabular cup it is necessary to estimate the shape of the ellipse. There are not currently any validated techniques for the measurement of ALO of micro BFX cups.

This study demonstrates the feasibility of hybrid THR in cats using a 12 mm BioMedtrix BFX acetabular cup. The use of this implant in a hybrid manner presents several advantages over cemented THR with the complication rate in this case series lower than reports of cemented THR. Long‐term radiographic follow‐up and greater case numbers are required to determine the long‐term outcome of this technique; however, these early results are promising.

## AUTHOR CONTRIBUTIONS

Lomas D, BVMed Sci (Hons), BVM, BVS (Hons), MANZCVS (Surgery), MRCVS: Study design, data collection, performed radiographic measurements, interpreted data, manuscript preparation and revision. Langley‐Hobbs S, MA, BVetMed DSAS (Ortho), DipECVS, FHEA: Study design, data collection, data interpretation, and manuscript preparation and revision. Parsons K, BVSc (Hons), PhD, DipECVS, FHEA: Study design, data interpretation, and manuscript preparation and revision. Barthelemy N, DVM, DipECVS: Study design, data collection, data interpretation, and manuscript preparation and revision. All authors provided a critical review of the manuscript and endorse the final version. All authors are aware of their respective contributions and have confidence in the integrity of all contributions.

## CONFLICT OF INTEREST STATEMENT

The authors have no conflict of interest to declare.

## Supporting information


Figure S1.
