# Supplementary material for: Medium‐term outcomes of hybrid total hip arthroplasty in cats: Cemented femoral stem and cementless acetabular cup in 17 hips (2020–2023)
Source: Vet Surg. 2025 Jun 5;54(6):1122–32. doi: 10.1111/vsu.14274 (PMC12344218; doi:10.1111/vsu.14274)
Supplement: Supplementary file 1 — Figure S1. [file VSU-54-1122-s001.pdf]

FELINE MUSCULOSKELETAL PAIN INDEX – short form

Please take some time to complete the following questions.
Please mark the circle that best describes your cat's ability to perform the following activities.

1. Jump up?

○	○	○	○	○
Normal	Not quite normal	Somewhat worse than normal	Barely, or with great effort	Not at all

2. Jump up to kitchen-counter height in one try?

○	○	○	○	○
Normal	Not quite normal	Somewhat worse than normal	Barely, or with great effort	Not at all

3. Jump down (how well and how easily)?

○	○	○	○	○
Normal	Not quite normal	Somewhat worse than normal	Barely, or with great effort	Not at all

4. Play with toys and/or chase objects?

○	○	○	○	○
Normal	Not quite normal	Somewhat worse than normal	Barely, or with great effort	Not at all

5. Play and interact with other pets?

○	○	○	○	○
Normal	Not quite normal	Somewhat worse than normal	Barely, or with great effort	Not at all

6. Get up from a resting position?

○	○	○	○	○
Normal	Not quite normal	Somewhat worse than normal	Barely, or with great effort	Not at all

7. Lie and/or sit down?

○	○	○	○	○
Normal	Not quite normal	Somewhat worse than normal	Barely, or with great effort	Not at all

8. Stretch?

○	○	○	○	○
Normal	Not quite normal	Somewhat worse than normal	Barely, or with great effort	Not at all

9. Groom himself or herself?

○	○	○	○	○
Normal	Not quite normal	Somewhat worse than normal	Barely, or with great effort	Not at all

NC STATE UNIVERSITY © 2020 North Carolina State University

NOTES TO ACCOMPANY THE FMPI-*sf* CLINICAL METROLOGY INSTRUMENT

Conditions of use:

- The FMPI-*sf* is designed as a Clinical Metrology Instrument (Questionnaire) for the assessment of Feline Musculoskeletal Pain. It can be used in clinical research studies, and also by practitioners for individual case assessment.
- Use of this questionnaire in a commercial setting (e.g. company funded clinical trials) requires the permission for use of the FMPI-*sf* under license from North Carolina State University.
- The FMPI-*sf* will be acknowledged in any publication or report by citing the appropriate reference.
- The FMPI-*sf* will be used only in the form presented here, and the format, wording and order of the questions and responses will not be changed.
- The FMPI-*sf* must not be given to others.
- The FMPI-*sf* must not be sold in any form.

The FMPI-*sf* is a questionnaire that has been designed through testing for readability, reliability and discriminatory ability. *Validity is a never-ending process, and further validity testing is ongoing.*

Instructions:

1. The following instructions should be read to owners by the operator each time the FMPI-*sf* is administered:
"This questionnaire asks you questions about your cat's ability to do various activities. Please read the questions and carefully check or fill in the appropriate circle. <u>Owners should be encouraged to answer all questions at every evaluation.</u>

2. Upon completion of the questionnaire, the owner should return the questionnaire to the administrator.

3. FMPI-*sf* scores are calculated by assigning whole integer scores from 0 to 4 to each question 1-9. Scores are assigned with 0 representing the option on the furthest left of the scale (the least affected option) and 4 representing the option on the furthest right of the scale (the most affected option).

4. The total FMPI-*sf* score is the mean score per question (total score/number of questions answered). Higher totals indicate more impairment.

5. If repeat FMPI-*sf* scores are acquired from an individual owner, they should not see their previous scores or responses prior to completing the questionnaire.

We welcome feedback on the FMPI-*sf*. Please contact Dr. Duncan Lascelles using: dxlascel@ncsu.edu
The Translational Research in Pain (TRiP) Program (*formally known as the Comparative Pain Research Laboratory*) is very grateful to Morris Animal Foundation, Novartis Animal Health, Boehringer Ingelheim Vetmedica Inc., Nexvet, Elanco and Zoetis for sponsoring the work that has led to the development and validation of the current version of the FMPI-*sf*.

 © 2020 North Carolina State University
